# A Review of Non-Invasive Sampling in Wildlife Disease and Health Research: What’s New?

**DOI:** 10.3390/ani12131719

**Published:** 2022-07-02

**Authors:** Anna-Katarina Schilling, Maria Vittoria Mazzamuto, Claudia Romeo

**Affiliations:** 1 Previously Royal (Dick) School of Veterinary Studies and Roslin Institute, University of Edinburgh, Easter Bush, Midlothian EH25 9RG, UK; annakatarinaschilling@gmail.com; 2 Haub School of Environment and Natural Resources, University of Wyoming, 1000 E. University Ave., Laramie, WY 82072, USA; mariavittoria.mazzamuto@uwyo.edu; 3 Department of Theoretical and Applied Sciences, University of Insubria, Via J.H. Dunant 3, 21100 Varese, Italy; 4 Istituto Zooprofilattico Sperimentale della Lombardia e dell’Emilia Romagna (IZSLER), Via Bianchi 9, 25124 Brescia, Italy

**Keywords:** non-invasive methods, wildlife species, wildlife pathogens, 3Rs, animal health, field sampling

## Abstract

**Simple Summary:**

The interest in wildlife research has increased in the last decades as more scientists work within a One Health framework that regards human, livestock and wildlife health as connected entities. To minimise the impact of research on wildlife, collecting samples with as little disturbance of the animals as possible is important. In our review, we assess the use of so-called non-invasive sampling and summarise which samples can be used successfully when carrying out research on wildlife diseases and health status. Our results show that interest in minimally invasive sampling has steadily increased since the 2010s. Topics able to employ these methods include disease research, but also stress and other hormone assessments, pollution studies, and dietary studies. At the moment, such methods are mainly used to collect samples from land mammals, however, they can also be used in a wide range of other animals. Ever more capable analytical methods will allow for an even wider use of such “animal-friendly” sampling methods.

**Abstract:**

In the last decades, wildlife diseases and the health status of animal populations have gained increasing attention from the scientific community as part of a One Health framework. Furthermore, the need for non-invasive sampling methods with a minimal impact on wildlife has become paramount in complying with modern ethical standards and regulations, and to collect high-quality and unbiased data. We analysed the publication trends on non-invasive sampling in wildlife health and disease research and offer a comprehensive review on the different samples that can be collected non-invasively. We retrieved 272 articles spanning from 1998 to 2021, with a rapid increase in number from 2010. Thirty-nine percent of the papers were focussed on diseases, 58% on other health-related topics, and 3% on both. Stress and other physiological parameters were the most addressed research topics, followed by viruses, helminths, and bacterial infections. Terrestrial mammals accounted for 75% of all publications, and faeces were the most widely used sample. Our review of the sampling materials and collection methods highlights that, although the use of some types of samples for specific applications is now consolidated, others are perhaps still underutilised and new technologies may offer future opportunities for an even wider use of non-invasively collected samples.

## 1. Introduction

In the last decades, awareness of the importance of studying and monitoring disease in wild animal populations has steadily grown. The need for a One Health approach that integrates wildlife, human, and domestic animal health into a single framework is now widely recognised [1] and has gained further attention due to the recent SARS-CoV-2 pandemic [2]. In addition to their relevance as a source of pathogens that may spill over to humans and domestic animals, many wild species are currently threatened by emerging diseases (e.g., white-nose syndrome in bats [3], chytridiomycosis in amphibians [4], or *Chlamydia* infection in koalas [5]), making wildlife disease research paramount from a conservation perspective [6,7]. Additionally, the need for a more complete understanding of disease circulation in natural populations has sparked the interest of many researchers who are now investigating those intrinsic factors affecting host–pathogen dynamics such as the host physiological and immunological status [8,9,10,11].

In parallel to the growing interest in wildlife disease, attention towards animal welfare in research has been growing since the ’60s, when Russell and Burch [12] proposed the 3Rs principle. It aims to replace animal use in research whenever possible, reduce the numbers of animals employed, and to refine the methods to limit their pain and distress. This principle has been incorporated in several legislations (e.g., [13]) and is now widely applied to laboratory animals. Ethical advances in wildlife research have been comparably slower, but the need for the development of non-invasive methods to safeguard animal welfare and avoid taking valuable animals from vulnerable populations is being advocated by several authors [14,15,16].

Wildlife disease and health surveys often rely on the opportunistic sampling of carcasses, and this is certainly an important resource for passive disease surveillance (e.g., [17,18,19,20] in the present Special Issue), but it can introduce relevant biases into epidemiological studies [21]. Random sampling of living individuals should therefore be preferred whenever researchers are interested in studying disease circulation in natural populations [22]. However, sampling living, free-ranging animals is a challenging endeavour, and biologists and veterinarians need to cope with several issues particular to the field that are not encountered when working with domestic or laboratory animals [23]. First and foremost, relevant constraints are related to logistics, as field work is often carried out in inaccessible locations, far away from laboratory facilities, making sample storage and transport a major concern. Another issue is related to capture and handling: capturing wild animals is normally work-demanding and indeed represents a source of distress for the animal, possibly altering physiological parameters [11] and posing ethical problems. 

Hence, a sampling method for wildlife should ideally be non-invasive, possibly require no trapping and/or handling to limit distress (although for some species this might be unavoidable) and be feasible in field conditions. Thanks to the growing interest in both wildlife diseases and health, the number of studies proposing alternative approaches aimed at making wildlife sampling easier and less invasive has increased. Such new approaches either focus on alternative methods to collect and use traditional samples, or on the validation of new sample materials that may represent a reliable alternative to traditional samples that would mostly require invasive collection (e.g., blood). Developing non-invasive methods for wildlife studies is likely to always be a multiple-step process in which samples are initially collected post-mortem or during direct contact with the animal to be able to establish whether a certain parameter or pathogen can be detected reliably. It then needs to be established how a sufficient amount of material can be collected from a live animal without direct interference and causing as little distress as possible. Finally, a further step may be necessary to prove that the results from the samples collected post-mortem and in live animals are actually comparable.

Our aim was to (i) analyse the publication trends on non-invasive sampling methods in wildlife health research and (ii) offer a review on the different samples that can be collected from wildlife non-invasively, what they are useful for and how they can be collected to survey for different pathogens or to investigate the physiological and immunological parameters in wild animals. Our intent in this review was to mimic a wildlife researcher, not necessarily a veterinarian, and possibly new to sampling for disease and/or physiology, starting to plan a new project involving field sampling, or are interested in using banked samples they have gained access to.

## 2. Materials and Methods

We searched for published literature on the Web of Science, Scopus, and Google Scholar platforms up until December 2021. Specifically, we used the following search string: (non-invasive OR noninvasive) AND (method* OR technique* OR tool* OR sampling) AND (wildlife OR “wild animals”) AND (disease OR pathogen* OR parasite* OR health). All search results were screened and those not relevant to the present synthesis were excluded. Additionally, we also used publications cited in the articles we found. Studies including post-mortem sampling were retained only when aimed at developing or validating non-invasive methods to be later applied on living individuals. Each paper was classified based on the parameters reported in Table 1. 

To assess the independence between some of the categories used to classify papers (Table 1), we performed a Pearson’s chi squared independence test. We used Monte Carlo simulation to obtain *p*-values without assuming asymptotically normal behaviour from small cell count (degrees of freedom do not go into the equation at any stage of the computation, so there is no reason to report them). Specifically, we tested the independence between “Biological material collected” and “Host’s taxon”, “Biological material collected” and “Topic”, “Detection method” and “Host’s taxon”. For these analyses, we excluded papers that were classified as “Reviews” and records with NAs.

## 3. Results

**Figure 1:**
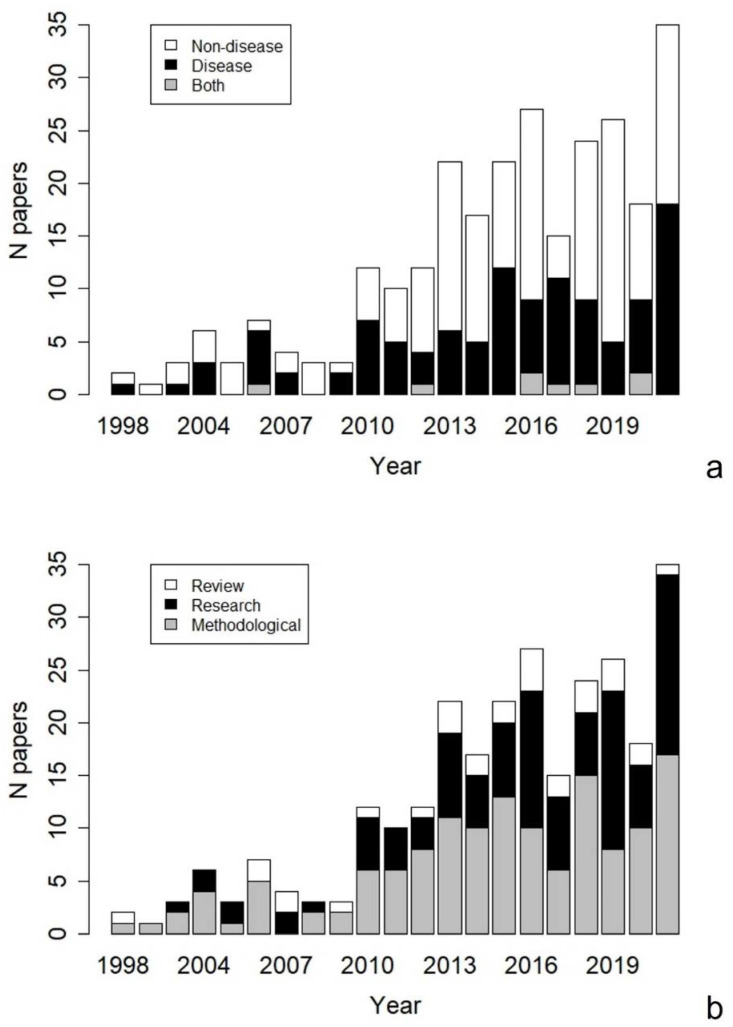
The number of articles on non-invasive sampling in wildlife published from 1998 to 2021 divided by general topic (**a**) and type (**b**). For a definition of categories, see Table 1.

Our search returned a total of 340 publications. After screening, we retained 272 papers spanning from 1998–2021 (median = 2016, Figure 1) and discarded the ones that were not relevant to this review. Thirty-nine percent of the papers were focussed on diseases, 58% on other topics, and 3% on both (see Figure 1a for a breakdown of the number of papers per year). We also classified papers based on their type: research articles (38%), methodological articles (51%), and reviews (11%) (Figure 1b).

**Figure 2:**
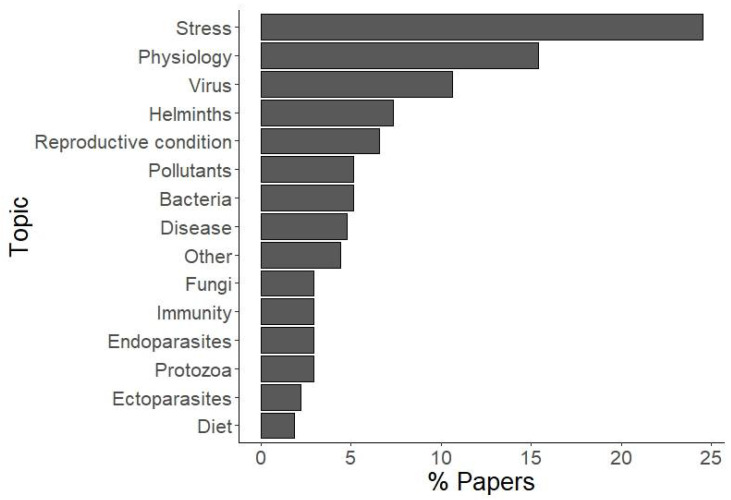
The percentage of published articles per topic. For a definition of categories, see Table 1.

Regarding the topic, “stress” was the one with the highest number of papers published (n = 67), followed by “physiology” (n = 42) and “virus” (n = 29). The other topics accounted for less than 10% each (Figure 2; Supplementary Materials Table S1).

The animal taxon most widely investigated was terrestrial mammals (Mammalia), accounting for 75% of all papers. The other taxa have been less studied: Aves 9%, Amphibia 6%, marine mammals 6%, Reptilia 3%, fish 1%, several taxa together <1%. Sixteen papers were classified as NA if the paper was a general review or methodological without a target species or taxon and were not included in the calculation of the percentage. Fifteen percent of the papers had captive wildlife as the host, mostly terrestrial mammals.

**Figure 3:**
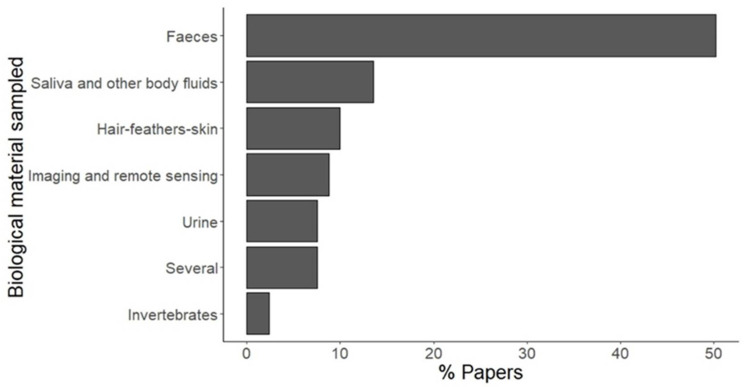
Th percentage of published articles by collected material. For a definition of categories, see Table 1.

Faeces were used as the biological sample in 50% of both the disease and non-disease papers. The second most used biological material was saliva and other body fluids (14%) (Figure 3; Table S2). 

The methods mostly used to collect biological materials were “Collection from the habitat” (39%) and “Trapping and handling” (36%) (Figure S1, Table S3). Finally, the detection methods mostly used were “Immunoassay” (39%), “Molecular method” (23%) and “Analytical chemistry” (13%) (Figure S2, Table S4).

Our analysis showed that “Biological material collected” was not independent from “Host’s taxon” (χ^2^ = 147.04, *p* < 0.001). In fact, there was a positive association between “Saliva and other body fluids” and “Amphibia”: this contributed 38% to the total Chi-square score, accounting for most of the difference between the expected and observed values (Table S5). “Biological material collected” was also not independent from “Topic” (χ^2^ = 249.08, *p* < 0.001): the test showed that “hair-feathers-skin” and “pollutants” were positively associated (22% contribution to the total Chi-square score; (Table S6). We found independence between “Host’s taxon” and “Detection method” (χ^2^ = 58.77, *p* = 0.136).

## 4. Discussion

We reviewed the scientific literature published in the last 25 years aimed at implementing non-invasive methods in wildlife disease research. Our analysis of the retrieved publications showed that the number of published articles regarding non-invasive sampling methods has been steadily rising since 2010. This suggests an increased ethical awareness of researchers and a growing need to comply with the more advanced ethical standards requested by society. Most of the publications were methodological articles aimed at describing new methods for the non-invasive collection of samples, but many of these were later applied in research studies. Several reviews were also retrieved, further testifying that the interest in non-invasive methods is nowadays consolidated.

The growing interest in non-invasive methods might also be partially related to the mounting evidence on the impact of acute stress on the physiological parameters and the immune response. Avoiding trapping and handling becomes in some cases a data quality requirement on top of the ethical necessity. This is further reflected by our analysis of the publication topic: stress was by far the most studied topic among those using non-invasive methods, followed by other physiological parameters (e.g., other hormones, body temperature, etc.) and viral diseases. Despite this, a considerable number of articles still relied on captures for sample collection, but this is, in our opinion, hardly avoidable when dealing with certain animal species or sample types or when the identity of the sampled individual is relevant to the research question. However, we believe that nowadays, capture protocols, can —and must— be defined to be as minimally invasive as possible. Additionally, concerning wild birds, many countries have continuous ringing programs in place that may represent an optimal opportunity for sampling, without resorting to additional captures specific for the research aim. 

Regarding the type of samples, faeces were by far the most frequently sampled material, followed by saliva and other body fluids and by hairs, feathers, or skin. This is at least partly due to the sheer number of articles on stress evaluation, as faeces are usually the material of choice when studying chronic stress in mammals, but it is also likely to be a consequence of the versatility of this biological material, which can be used for several purposes (see below). Likewise, immunoassays are usually employed to quantify hormones, or their metabolites and they were indeed the most frequent technique applied on samples in the retrieved papers. These were followed by molecular methods, which nowadays are a fundamental tool in pathogen detection, especially viruses and bacteria. Finally, the vast majority of the publications regarded terrestrial mammals, which is unsurprising, considering that such taxonomic bias is well-recognised and common to several fields of wildlife biology [24,25,26,27], disease ecology included [28,29].

Below, we will present an overview of the reviewed techniques and methods for collecting samples non-invasively from wildlife, both for disease and health or physiology surveys.

### 4.1. Faeces

Thanks to their versatility and ease of sampling, faeces are undoubtedly the most widely used material for the non-invasive monitoring of diseases and health in wildlife. This was confirmed by our analysis on the published literature where 126 articles using faecal samples were retrieved.

Regarding their use, faecal samples are of course essential for endoparasite investigations on living animals as they can be used to detect helminth eggs or larvae and coccidian oocysts that are shed with faeces. This is still mostly conducted through traditional copromicroscopy and morphological identification of the isolated specimens [30,31,32,33]; molecular approaches can also be applied to identify endoparasite life stages at the species level, since this cannot always be achieved through morphology alone. The extraction, amplification, and sequencing of parasite DNA using species-specific or group-specific primers can be carried out directly from faecal material (e.g., *Echinococcus multilocularis* in coyotes *Canis latrans* [34]), but most often faeces are first processed to allow for the isolation of helminth eggs and larvae [35,36,37], or oocysts [38,39] to achieve better results. Moreover, constant advances in genomics and a greater affordability of high-throughput sequencing make metabarcoding a promising tool for the simultaneous screening of all parasitic DNA present in a faecal sample (reviewed in [40]). 

These same molecular methods can also be employed to detect viral nucleic acids from faeces, avoiding invasive blood sampling. Faecal samples were used to screen for a wide variety of viruses belonging to several families (e.g., [41,42,43]), sometimes of zoonotic (e.g., Lassa virus in rodents [44]) or conservation relevance (e.g., peste des petits ruminantes in wild ungulates [45]). Specific molecular analyses (i.e., protein misfolding cyclic amplification, PMCA) on faeces have also been used successfully to detect prion diseases such as chronic wasting disease, CWD, in ungulates [46]. Faeces are also routinely used to screen for pathogenic bacteria by standard microbiological techniques or molecular identification (*Clostridium perfringens* [47]; *Rickettsia felis* [48]; *Salmonella enterica* serovar. Enteritidis [49] and Typhimurium [50]; *Mycobacterium bovis* [51]), and nowadays, high-throughput metagenomic techniques allow us to characterise the whole gut microbial community composition at once including the pathogenic bacteria from a single faecal sample [52,53]. Of course, in a One Health context, faeces from wildlife can also be used to investigate antimicrobial resistance (AMR), either by screening for AMR genes through molecular methods, or by culture and phenotypical methods (e.g., [54] on *Staphilococcus aureus* in lemurs).

Other than for pathogen detection, faecal samples represent a source of data on the general health status of wildlife, as they can be used to assess several physiological parameters, the presence of pollutants, and the diet. The metabolites of steroid and thyroid hormones are excreted with faeces, allowing for their non-invasive quantification through specific immunoassays (reviewed in [55,56,57]) or, less frequently, through mass spectrometry [58]. It is important to note that the species-specific validation of hormonal immunoassays is crucial to obtain reliable results since hormone metabolism and excretion may greatly vary, even within the same genus [59]. However, glucocorticoid metabolites have already been used as a measure of chronic stress and assessed from faeces in a variety of mammalian species [60,61,62,63,64,65,66,67], birds [68,69,70] as well as reptiles [71]. Likewise, immunoassays on faeces allow for the quantification of the faecal metabolites of sexual steroids [72,73,74,75] and can be used, for instance, to monitor the reproductive cycles and pregnancy in endangered species (wombats species [76]; southern white rhinoceros *Ceratotherium simum simum* [77]; collared peccary *Pecari tajacu* [78]; tigers *Panthera tigris* [79]). Finally, faeces can also be used to study the diet of vertebrates (either by traditional microscopy or metagenomic techniques: [80,81,82,83]) and to screen for the presence of pollutants or toxic compounds by analytical chemistry [84,85].

For studies at the population-level or disease surveillance, fresh faeces can be collected directly from the habitat (e.g., from pastures, dwellings, latrines, or under nesting sites, depending on the species) without any direct contact with the animal (wild cats *Felis silvestris silvestris* [86]; badgers *Meles meles* [87]; Gentoo penguins *Pygoscelis papua* [88]; several Carnivora and Artiodactyla species in Brazil [89]; Amur leopards *Panthera pardus orientalis* [90]; wolverines *Gulo gulo* [42]). In the case of large mammals, GPS-telemetry might be useful to monitor animal movements and locate areas where faeces are most likely to be found, as it was conducted by Van der Goot and colleagues [77] on southern white rhinoceros or by Di Francesco and colleagues [91] on wolf (*Canis lupus*) packs. For flying or arboreal species (e.g., the lesser horseshoe bat *Rhinolophus hipposideros* [92]), plastic sheets can be placed under nests or roosting sites to facilitate sample collection.

However, when information at the individual-level is needed, researchers may need to resort to observation from a distance to collect fresh faeces as soon as an individual defecates (Upland geese *Chloephaga picta leucoptera* [69]; Barbary macaques *Macaca sylvanus* [93]; red deer *Cervus elaphus* [94]; North Pacific grey whales *Eschrichtius robustus* [75]) or to captures, especially in the case of small birds and mammals (southern pied babblers *Turdoides bicolor* [95]; Muridae and Tupaide in Borneo [96]; Namaqua rock mice *Micaelamys namaquensis* [65]; desert gerbils [66]). Serres-Corral and colleagues [67] applied an interesting technique to individually mark faeces from lions (*Panthera leo*) held in captivity, making use of non-toxic coloured waxes administered with feed. However, applying this method on free-living individuals might prove more challenging. 

Once collected, faecal samples may be generally kept safely at room temperature for a limited amount of time, but will then need to be stored appropriately, depending on the specific aim of the study. For example, when carrying out standard copromicroscopy to identify helminth eggs or larvae, to preserve their morphology, faeces are best stored dry at 4 °C and processed within a few days, while freezing is not recommended [97]. On the other hand, storage at −20 °C is needed in the case of genetic analyses or immunoassays on hormones [98]. Additionally, a certain sterility of the sample is sometimes required, for instance, Knutie and Gotanda [52] developed a cheap and easy-to-build device to collect faeces from birds in sterile conditions for microbiome studies. It is also best to avoid contamination with urine when analysing faeces for hormone metabolites because metabolites are also excreted with urine (see below), potentially leading to an overestimation of hormone levels [55,57,59]. Finally, it is worth mentioning that the extraction of nucleic acids from faeces can be challenging compared to other samples due to the presence of inhibiting substances; as a consequence for molecular analyses, specific extraction protocols are required [99,100]. Moreover, when using molecular tools to survey for parasite life stages, additional sample processing steps aimed at breaking egg walls or larvae such as magnetic beads or sonication are needed in order to be able to efficiently extract and amplify DNA (e.g., [101]). 

### 4.2. Urine

Urine was used as the sample material in 19 publications included in this review, 14 of these were studies on primates. Beyond the standard clinical dipstick, urine can be used in the detection of pathogens and to assess the physiological parameters associated with stress, reproductive status, inflammation, and metabolic status. 

Urine has been used to detect helminths, for example, the eggs of the nematode *Stephanurus dentatus* that parasitize the urinary tract of wild boars (*Sus scrofa*, [102]) and the antigens of *Taenia serialis* in the urine of gelada monkeys (*Theropitecus gelada*, [103]). Bacterial pathogens with a tropism for kidneys such as zoonotic *Leptospira* spp. have been successfully detected in urine as part of the monitoring and surveillance efforts [104]. The detection of some viruses (e.g., paramyxoviruses in African fruit bats) or antiviral antibodies against simian T-lymphotrophic virus type 1 (STLV-1) in chimpanzees (*Pan troglodytes*) is also possible [105,106,107]. Urine can also be used for hormonal assessment. For example, stress steroids and their metabolites excreted with urine have been successfully quantified through immunoassays in several mammalian and amphibian species [108,109,110,111,112,113,114]. Sexual steroid hormone levels (or their metabolites) can also be assessed from the urine and have thus been used to investigate the reproductive status in mammals (giant pandas *Ailuropoda melanoleuca* [115]; chimpanzees [116]) as well as amphibians (reviewed in [112]). Other hormones that can be isolated from urine are oxytocin [117] and thyroid hormones (reviewed in [56]). The cell-mediated immune response has been also monitored from urine in a range of primates by the quantification of neopterin, a biomarker that increases when an acute inflammation is present [114,118,119,120,121,122,123]. Metabolic state has also been assessed by measuring urine triiodothyronine in macaques [114], by determining C-peptide and ketone bodies in orangutans [124], and by assessing the nitrogen:creatinine ratio in wild moose [125,126]. 

In arboreal mammals, urine can often be collected from the ground or leaves immediately after spontaneous urination or by spreading plastic sheets under known roost or sleeping sites [103,105,106,107,108,114,115,116,117,118,119,120,121,123,124]. In cold climates, it may be possible to collect naturally frozen urine samples from the snow, however, the dilution effect of the snow needs to be taken into account for analysis [125,126]. If mammals are anaesthetised for other procedures, it is likely that they will urinate during recovery, so sample collection can be attempted in this phase [123]. In amphibians or small mammals, collection during handling is often possible, either by stimulating urination by massaging the belly or by using small suction devices employing capillary forces, which are applied to the cloaca or end of the urethra [104,109,110,112,113]. After collection, the two main ways of storing urine samples are either immediate freezing, and afterwards limiting the freeze–thaw cycles to a minimum, or the use of filter papers. If fresh urine for neopterin analysis is to be kept for longer periods of time at room temperature, biocidal preservatives can be added to reduce its deterioration [118].

Similarly to faeces, for surveillance efforts in a population, the identification of individuals may not be that relevant; however, it remains important to be aware if there is a risk of contamination of the samples with urine from other cohabitating species than the target one [107]. Finally, the volume of urine samples is often limited and contamination with faeces and soil or exposure to sunlight can have an impact on the analytical results, depending on the component assessed [114,117,118,119]. 

### 4.3. Saliva and Other Body Fluids

The use of saliva and other body fluids (exhaled breath condensate (blow), breath, lacrimal fluid, skin mucus) was described in 34 papers included in this review. This sample category was widely used on amphibians (in 15 out of the 22 publications retrieved for the taxon), where skin mucus represents an important resource for the non-invasive monitoring of diseases and physiological parameters.

The potential uses of saliva are manifold and include stress monitoring, pathogen detection, and antibody detection. Stress monitoring is possible both by cortisol measurement [127,128] and by detecting a suppression in secretory Immunoglobulin A, an indicator of chronic stress [129], given that baseline values are established for the species and population under investigation. Both bacterial pathogens excreted orally such as *Mycobacterium bovis* in Australian brushtail possums and oral microbiomes can be assessed by extracting bacterial DNA and RNA from saliva [130,131]. A wide range of viruses can be isolated from saliva including economically relevant viruses such as foot-and-mouth disease virus [132,133], and classical and African swine fever [134,135] as well as viruses of conservation concern such as the elephant endotheliotropic herpesvirus [136]. All examples of virus isolation from saliva that we found in this review were conducted in mammals [132,133,134,135,136,137,138,139]. Antibody detection in saliva has, for example, been attempted for *M. bovis* antibodies in wild boar [140]. The collection of saliva samples can be carried out directly by rolling a cotton swab over the oral mucosa of a restrained animal [127,128,131,136,137], or indirectly by using artificial baited objects or natural objects from the animals’ environment that they routinely interact with. An artificial object that can be attractive to a range of inquisitive mammalian species including, for example, suids and primates, are ropes covered in bait [133,134,135,137,140]. Baited salt licks [132], interactive enrichment toys [139], and WaxTags^®^[130] are other objects that encourage animals to lick them and leave saliva behind for collection. Natural objects include all items that a specific species tends to chew and discharge, for example, leaves in some primates [138]. When choosing between direct and indirect collection, it needs to be considered that while direct collection allows us to assign a sample to an individual with great certainty, it also requires this animal to be physically or chemically restrained or long-term habituated and trained for sampling [128]. Indirect sampling removes the need to handle an animal, but requires observation (camera or direct) of the collection object [138], or additional DNA assessment to establish the individual that a sample came from. Choosing a safe and effective object for saliva collection for a species requires detailed knowledge of its behaviour and preferences. To optimise sampling, pre-trials with different objects are often necessary. The risk of interactions of non-target species with the object, objects becoming unretrievable or being swallowed also need to be managed [130,135,137]. Some objects such as cotton ropes may contain natural PCR inhibitors that need to be considered when samples are processed [135,137]. Generally, in saliva samples, it needs to be considered that the oral microflora can be sensitive to external conditions [131] and that low pathogen excretion can occur, potentially reducing the sensitivity of surveillance efforts using saliva [134]. 

Blow collection has been described for a range of marine mammals including, for example, humpback whales (*Megaptera novaeangeliae*), harbour porpoises (*Phocoena phocoena*), and belugas (*Delphinapterus leucas*) [141,142,143]. It has been used for bacterial detection [144], respiratory microbiome analysis [145], and viral detection [142] as well as volatile organic compound analysis [146] and cortisol and the detection of other steroid hormones [141,143]. Blow can be collected in a variety of vials, bags, and dishes, either held in the hand if it is possible to get close to the animal or mounted on a pole to be able to work from a greater distance [141,142,146]. Petri dishes tend to allow for the collection of the largest volumes of fluid and can be readily covered in materials and membranes to enhance sampling based on the individual research question [141,143]. A factor to consider when planning to use blow samples is that the sample volume and dilution cannot be controlled, and that the confirmation of the presence of sufficient organic material for analysis may need to be included in any experimental design [141,143].

Breath collection in mammals has been trialled for use in indirect pathogen detection, as the presence of certain pathogens such as *M. bovis*, MAP or *Brucella spp.* can lead to characteristic changes in the composition of volatile organic compounds in breath [147]. As the collection is currently only possible using a complex mask that needs to be tightly fitted to the animal’s nose, it can only be used in habituated and fixated individuals [147], which will limit its applicability to wildlife. 

Lacrimal fluids have been assessed as a low impact sample to detect cortisol in harbour seals [148]. Animals need to be restrained for sampling and a cotton swab inserted into the conjunctival sac for 15 s. The cortisol concentration in lacrimal fluid did show good correlation with values measured in blood in the same animals, but the measurement range for this new sample type would need to be established for each species it is to be used on [148]. 

Skin mucus has been collected from amphibians [111,149,150,151,152,153,154,155] and fish [156,157,158]. In amphibians, the main focus of studies utilising skin mucus has been the detection of the fungal pathogen *Batrachochytrium dendrobatis* [111,150,151,153,154,155]; however, wider investigations of the skin microbiome [149] and cortisol detection [152] are further uses for this material. The sensitivity of pathogen detection in skin mucus may be limited when the intensity of infection is low [151]. In fish, skin mucus is mainly used for stress assessments, both by isolating cortisol and its metabolites [157] and by assessing the oxidative stress using F2-isoprostanes [156,158]. Mucus collection has only been described during direct handling, either by swabbing or scraping of the skin [151,152,156,158]. This may in itself alter the cortisol excretion, both systemically and locally in the skin [152]. A further method for the minimally invasive assessment of steroid hormones in amphibians and fish is the immersion of caught individuals in a clean tank of water for a predetermined period of time, after which the animal is moved and excreted hormones can be extracted from the water [159,160,161,162]. These assays can also be used on environmental water samples; however, ambient cortisol and metabolites as well as steroid hormones may persist in the environment to varying degrees and limit the information that can be gathered about an individual in, for example, a pond [162]. 

Overall, saliva and other body-associated fluids have great potential both in health and welfare surveillance (microbiome studies, stress assessment) and in pathogen monitoring. However, being able to collect samples that can clearly be assigned to an individual animal without handling it remains a challenge as well as the standardisation of the volume and dilution of samples collected with more hand-off methods.

### 4.4. Hair, Feathers, and Skin

The collection and analysis of hair, feather, or skin samples were mentioned in 25 papers included in this review. These sample materials are the preferred media for investigating contaminants; indeed, out of the 16 retrieved publications aimed at detecting pollutants or medicine residuals, 11 relied on either hair or feathers.

Hair samples are used for pollutant and mineral detection in a wide range of mammals, from bats to polar bears [163,164,165,166,167,168]. Similarly, hair can be used to detect medicine residuals [169]. Further uses are the determination of cortisol concentrations for stress monitoring [167,170,171,172,173,174,175,176] and the assessment of steroid hormone concentrations [177]. Some pathogens, for example, adenovirus and squirrelpox virus in Eurasian red squirrels (*Sciurus vulgaris*) [178] and protozoa *Leishmania infantum* can be isolated from hair samples [179,180]. A final use linked to wildlife health is the assessment of nitrogen, carbon, and sulphur composition in the hair to gain insights into the animals’ diet including periods of negative energy balance [181,182]. Hair is therefore a very versatile and valuable source of information, however, while a collection is possible without direct contact with the animal, for example, by using hair traps, barbed wire, or collection from known rubbing trees [164,181], most studies included in this review gathered their samples either during direct handling of the physically or chemically restrained animal [168,170,171,177,178,181,182] or from carcasses during post-mortem examination [163,164,165,166,167,169,172,176,178,179,180,181,182]. This implies that further research into low-impact collection methods from live, free-roaming animals is necessary to use this sample type to its full potential. Once the sample is collected, analytical and diagnostic methods for minerals, pollutants, residuals, diet, and pathogens are usually well-established. It does need to be established for each species, though, whether the correlation between the blood levels and levels in hair is reliable, as it may be poor in some species [168]. With regard to pathogens, the location a pathogen takes after infection needs to be taken into consideration to determine whether an animal is infected with a certain pathogen or if the pathogen may just be present on its fur as a contaminant. To use hair samples for cortisol determination, it needs to be considered that in some species, age, pregnancy, colour, body region, sex, and season influence cortisol levels [170,174,175], and that local cortisol production in the hair follicle may occur, in which case the hair cortisol concentration may not offer information on the HPA-axis activity [170]. A final factor to consider when designing studies using hair samples is the number of hairs needed to produce reliable results, as too small a sample may result in false negatives, while too large a sample may cause inhibition during analysis [178]. 

For feathers, our search only flagged up publications using them for the detection of heavy metal pollution (As Cd, Cu, Hg, Ni, Pb, Se, Zn) or mercury alone (Hg) [183,184,185,186,187]. However, based on the experience with hair, it may eventually be possible to use feathers for the detection of other contaminants such as medicine residuals. While one study relied on feathers collected post-mortem [183], others have used the plucking of feathers during physical restraint and handling [184,187]. It has, however, been shown that meaningful results can also be achieved by collecting clean, freshly shed feathers from nest boxes, still allowing a certain level of information of which animal a feather came from, or at least narrowing the pool down to the breeding pair and their offspring [186]. Disturbance to the nest needs to be weighed against handling stress to determine which of the two collection methods actually has less impact on the species under investigation in each study. 

Skin samples of marine mammals can be used for trace element detection (Al, V, Cr, Mn, Cu, Zn, As, Se, Rb, Sr, Mo, Cd, Pb), but only if a full biopsy is collected, which, depending on size, can actually be an invasive procedure [188]. Truly minimally invasive samples of dead superficial skin cells collected with combs can be used for ectoparasite detection, for example, *Antarctophthirus microchir* in American sea lions (*Otaria flavescens*) [189]. Skin scrapings are commonly used on several species for *Sarcoptes scabiei* detection. However, depending on the handler experience and technique used, they may reach a depth where they draw blood and are no longer truly minimally invasive. Fraser and colleagues [190] compared the efficacy of PCR on skin scrapings and on less-invasive skin swabs for *S. scabiei* detection in wombats (*Vombatus ursinus*). Although they showed that PCR has a higher sensitivity than standard microscopy for ectoparasite detection, they concluded that PCR on swabs is less accurate [190]. For reptiles, the detection of mercury in skin scutes has been described [191]. It would be of interest to assess whether the shed skins of reptiles could be used with similarly reliable results. 

### 4.5. Imaging and Remote Sensing

This sample material class included 22 articles that mostly studied the physiological parameters of wild mammals (terrestrial and marine), birds, or reptiles. However, a few studies have also used imaging, observation, and remote sensing to investigate the parasites and health conditions. 

One of the tools used in most of the studies was infrared thermography (IRT). Thanks to the development of new and lower cost portable systems, IRT has become a non-invasive technique that allows for the precise measurement of infrared radiation, and hence spatial variation in body surface temperature, it can be used at less than one metre to investigate specific body regions, or from a distance up to hundreds of meters when just the detection of an animal in the wild is desired. The physiological parameter mostly recorded with IRT clearly was the body temperature of wildlife in the wild and in captivity. While some studies have focused on the study of thermoregulation mechanisms (e.g., [192,193,194]), others have used body temperature as a proxy for health, body condition, and the metabolic state of an animal (e.g., [195,196,197]). For example, Burns, McCafferty, and Kennedy [192] studied the nesting thermal biology of wild leatherback turtles (*Dermochelys coriacea*), avoiding any interference or stress in this reproductive phase thanks to the use of thermal imaging. Researchers were able to study the spatial and temporal variation in the body surface temperature through all phases of nesting behaviour, and to estimate the relationships between body temperatures and the environmental conditions of the beach. It is important to highlight that those studies using IRT need to consider some important parameters to record accurate temperatures and avoid errors (e.g., variation in emissivity, evaporative cooling, radiative heating of the coat) [198]. This was also highlighted by Horton and colleagues [193], who paired the technology of IRT with that of unoccupied aerial systems (drones) to document the blowhole and dorsal fin skin temperature, respiration rate, and heart rate of adult and calf humpback whales (*Megaptera novaeangliae*). Few attempts have been made to use IRT in the detection of disease in wild mammals [mange [199]; rabies [200]; foot-and-mouth disease [201]]. However, the latter two studies were carried out in a controlled environment in captivity. Arenas and colleagues [199] tested IRT to diagnose inflamed skin infected by sarcoptic mange (*Sarcoptes scabiei*) in wild Spanish ibex (*Capra pyrenaica*). Unfortunately, visual observation resulted in being more effective than IRT due to the low accuracy of the thermal image at distances greater than 100 m. 

Since *S. scabiei* results in visible hair loss and affects a broad host range, another tool that has been successfully used to detect mange in wild canids are camera traps [202,203]. However, the distance at which the animal is detected by the camera, hence the resolution of the image, could be a limitation if this tool is to be used for early diagnosis to prevent epidemics of mange that could otherwise have high morbidity and mortality rates. Camera traps can be used to detect any health condition that affects the look of an animal in the wild. For example, Lacroux and colleagues [204] successfully investigated the occurrence of facial dysplasia in wild forest olive baboons (*Papio anubis*) in Uganda as a suspected alteration of embryonic development due to the use of pesticides in crops at the border of their habitat.

A more recent paper showed the use of a common survey tool for bats as a surveillance method for white-nose syndrome (WNS) [205]. Thanks to the collection of bat echolocation sound, the authors developed site-specific prediction models for bat activity accounting for seasonal and annual temperature variation prior to the known occurrence of WNS. These models were then used to monitor changes in bat activity that may signal the potential presence of WNS. While this seems to be an effective method to detect WNS, the authors highlight the need to collect baseline (pre-WNS) data across several years and sites and then look for bat activity in excess of the predicted range during winter months, followed by bat activity below the predicted range during the following spring and summer. As a consequence, this approach cannot be applied to populations that might already be infected and is simple to apply only if passive autonomous recording units are deployed. 

### 4.6. Other 

A few papers have used other animals as either a detection or collection tool. This is the case of the sample class “Invertebrates”, where researchers have used blood sucking invertebrates to collect and then analyse the blood of the target wildlife species (six papers). It must be noted, however, that although these studies considered collecting blood samples through invertebrates as non-invasive, this method is lethal to the invertebrates used. Two studies used leeches to collect blood from mammals. Kvapil and colleagues [206] used medicinal leeches (*Hirudo medicinalis*) as an alternative to more complex and invasive methods for blood sampling for preventive medicine and epidemiological studies in zoo animals looking for antibodies to tick-borne encephalitic virus. Alfano and colleagues [207] collected leech-derived iDNA (terrestrial leeches: *Haemadipsa picta, H. zeylanica*) in the forests of the Malaysian Borneo to detect and identify known and novel mammalian viruses. In this study, iDNA was also paired with eDNA from waterholes to study wildlife viral diversity and to detect novel potentially zoonotic viruses prior to their emergence. Thomsen, Voigh, and colleagues [208,209] tested the use of kissing bugs (Reduviidae; Triatominae; Heteroptera) as a gentler system to collect blood to detect rabbit haemorrhagic disease virus in domestic rabbits [209] and in captive primates [208]. Hoffmann and colleagues [210] assessed the feasibility of the fly-based surveillance of wildlife infectious diseases, specifically for adenoviruses (family Adenoviridae), in wild-living vertebrates in a tropical rainforest in Côte d’Ivoire. They concluded that this method could probably be used to detect the genetic material of wildlife infectious agents causing wildlife mass mortality in pristine areas. However, they also highlighted that characterising the genetic diversity of wildlife infectious agents through fly-based monitoring may not be cost-efficient. Other invertebrates such as engorged ticks collected from wild hosts may be used to indirectly screen for pathogens, as was carried out, for instance, by Zechmeisterova and colleagues [211], who used engorged ticks to detect parasitic protozoa in the Iberian lizard (*Lacerta schreiberi).*

The detection of viral infections can also be conducted collecting samples from the environment where the targeted wildlife live. The study previously mentioned by Alfano and colleagues [207] paired the iDNA analyses with those of the eDNA collected from waterholes in Tanzania and Mongolia. The indirect transmission of beak and feather disease virus, which is of global concern and can cause lethal infections, was tested by Martens and colleagues [212] using nest swabs. They provided novel insights into the potential role of nest cavities and other fomites in the indirect transmission of this virus and possibly other pathogens, and offered a non-invasive method for the surveillance of pathogens in wild bird populations.

The use of odour-detection dogs was evaluated as the methodology to locate faecal samples that were then analysed to detect parasite infections. Detection dogs resulted in being more efficient than humans in detecting scat samples [213] and allowed for the collection of more than 600 cervid scats in the study by Teixeira and colleagues [39] in Brazil with the aim of detecting and genetically characterising the infections by *Cryptosporidium* in brockets (*Mazama gouazoubira*). Curry and colleagues [214] tested, for the first time, the use of sniffer dogs in the medical diagnoses of wildlife species. They investigated the reliability of a trained dog for pregnancy detection in polar bears *Ursus maritimus.* The dog had a reduced sensitivity in testing versus training and the authors discussed the possible causes. Moreover, the authors suggest that it is likely that many unique cases of condition are required to sufficiently train an odour-detection dog, which may be prohibitive in wildlife studies when sample sizes are liable to be limited.

### 4.7. Additional Considerations

We designed our search to mimic a wildlife researcher, possibly new to sampling for disease and/or physiology, starting to plan a new project involving field sampling or interested in using banked samples they have gained access to. In particular, minimally invasive samples are often collected from wildlife without a specific goal in mind and stored until it is possible to acquire funding or connect to colleagues with the right skills and equipment to analyse them further. This approach may seem alien to researchers used to working in laboratory-based settings; however, it is extremely important in wildlife research due to the often challenging field conditions that make every sampling occasion an opportunity to be seized. Therefore, we designed our search in the broadest way we could think of, attempting to capture as many publications presenting or discussing minimally invasive methods as possible. However, we soon realised that our search missed several publications that might have been of interest. For instance, even some of our own papers using non-invasive methods were not retrieved (e.g., [215,216]). Other articles deserving of attention were pointed out to us by peer-reviewers such as a study using ticks to survey for pathogens in wombats [217], another one describing the use of a cytology cell sampler to collect samples from lesions in marine mammals [218], or yet another describing the use of drones to collect blow from them [219]. We were also made aware of the opportunity to detect viruses and cortisol from bird feathers [220,221] or to use pellets from birds of prey or owls for microbiological studies [222,223]. This led us to reflect on why all of those articles had been missed and we concluded that in most cases, neither we nor our colleagues had made it sufficiently explicit that the research employed non-invasive methods (i.e., we had not included the term in the title, keywords or the abstract, and sometimes not even in the full text). This may reduce the opportunities to inform future projects and may slow down the process of employing minimally invasive methods to their full potential. We would therefore like to use this final paragraph to encourage the research community using non-invasive methods on wildlife to tag this clearly and make the information easily accessible to all those interested.

## 5. Conclusions

Our review of the literature addressing non-invasive methods for the disease and health monitoring of wildlife has highlighted the growing interest of the research community in sampling free-ranging animals non-invasively, with as little interference as possible. However, we believe that there is still much room for improvement, for instance, saliva and urine have been proven to be effective methods for the detection of some pathogens, but still appear underutilised, perhaps because collection can be more challenging compared to other mediums. However, our review highlighted that some specific methods and novel devices for their sampling have already been developed. Additionally, new opportunities for the development and implementation of non-invasive sampling may arise in the future following advances in rapidly evolving fields such as molecular biology or imaging and remote sensing technologies. Finally, we would like to stress again that, although trapping and handling might be still unavoidable in some cases, researchers should always employ rigorous protocols aimed at minimising distress for the animal. A responsible use of wildlife in research, in line with biodiversity conservation efforts, must make the further development of non-invasive sampling methods and a comprehensive use of all samples available for as many types of analyses as possible a priority. We sincerely hope that the trends for the increased use of non-invasive methods as identified in this review continues.

## Figures and Tables

**Table 1:** The categories used to classify articles included in the present review.

Topic		Type of Article	Host’s Taxon	Material Collected	Collection Method ^1^	Detection Method ^2^
Disease		Research ^5^	Amphibia	Faeces	Collection from habitat	Analytical chemistry
	Bacteria	Method ^6^	Aves	Hair-feathers-skin	Hair trapping	Coprological method
	Disease	Review	Fish	Imaging and remote sensing	New device	Coprological method combined ^11^
	Ectoparasites		Mammalia	Invertebrates ^8^	Other animals ^9^	Imaging
	Endoparasites		Marine mammal	Saliva and other body fluids	Post-mortem ^10^	Immunoassay
	Fungi		Reptilia	Urine	Trapping and handling	Molecular method
	Helminths		Several ^7^	Several ^7^	Visual	Molecular method combined ^12^
	Protozoa				Several ^7^	Other ^3^
	Virus					Several ^7^
	Other ^3^					
Non-disease						
	Diet					
	Immunity					
	Physiology ^4^					
	Pollutants					
	Reproductive condition					
	Stress					
	Other ^3^					
Both						

^1^ Method used to collect the sample; ^2^ Method applied on the sample for pathogen detection and/or health parameters assessment; ^3^ Topic not included in any of the above categories (e.g., prion diseases); ^4^ Hormones other than stress steroids, metabolism, body temperature, and other physiological parameters; ^5^ Research articles applying non-invasive sampling methods; ^6^ Research articles defining, testing, or validating new non-invasive methods; ^7^ Two or more of the above categories included; ^8^ Blood-sucking invertebrates used to collect blood from target species; ^9^ for example, blood-sucking invertebrates, sniffer dogs; ^10^ roadkill, found carcasses, or hunted animals used for non-invasive method validation; ^11^ Coprological method combined with immunoassay, molecular method or observation; ^12^ Molecular method combined with microscopy or culture.

## Data Availability

No new data were created or analyzed in this study. Data sharing is not applicable to this article.

## Supplementary Materials

The following supporting information can be downloaded at: https://www.mdpi.com/article/10.3390/ani12131719/s1. Table S1. Number and percentage of papers published per topic. Table S2. Number and percentage of papers published per biological material collected (21 NA removed). Figure S1. Percentage of published articles classified based on method used to collect the samples. Table S3. Number and percentage of papers published per biological material collection method (26 NA removed). Figure S2. Percentage of published articles classified based on the detection method. Table S4. Number and percentage of papers published per detection method (18 NA removed). Table S5. Percentage contribution to the Chi-squared independence test biological material sample collected—Host’s taxon. Table S6. Percentage contribution to Chi-squared independence test Topic—Biological material sample collected.
