# Supplementary material for: A Review of Non-Invasive Sampling in Wildlife Disease and Health Research: What’s New?
Source: Animals (Basel). 2022 Jul 2;12(13):1719. doi: 10.3390/ani12131719 (PMC9265025; doi:10.3390/ani12131719)
Supplement: Supplementary file 1 [file animals-12-01719-s001.zip › animals-1772699-supplementary.pdf]

Supplementary Material

Review

A review of non-invasive sampling in wildlife disease and health research: what's new?

Anna-Katarina Schilling [1,†], Maria Vittoria Mazzamuto [2,3,†] and Claudia Romeo [4,*]

[1] Veterinarian practitioner; annakatarinaschilling@gmail.com
[2] Haub School of Environment and Natural Resources, University of Wyoming, 1000 E. University Ave, Laramie, WY, 82072, USA; mariavittoria.mazzamuto@uwyo.edu
[3] Department of Theoretical and Applied Sciences, University of Insubria, Via J.H. Dunant 3, 21100, Varese, Italy
[4] Istituto Zooprofilattico Sperimentale della Lombardia e dell'Emilia Romagna (IZSLER), via Bianchi 9, 25124, Brescia, Italy; claudiarosa.romeo@izsler.it
† these two authors equally contributed
* Correspondence: claudiarosa.romeo@izsler.it

Table S1. Number and percentage of papers published per topic.

**Topic**	**N**	%
Stress	67	24.63
Physiology	42	15.44
Virus	29	10.66
Helminths	20	7.35
Reproductive condition	18	6.62
Pollutants	14	5.15
Bacteria	14	5.15
Disease	13	4.78
Other	12	4.41
Fungi	8	2.94
Immunity	8	2.94
Endoparasites	8	2.94
Protozoa	8	2.94
Ectoparasites	6	2.20
Diet	5	1.83

Table S2. Number and percentage of papers published per biological material collected (21 NA removed).

**Biological material collected**	**N**	**%**
Faeces	126	50.00
Saliva and other body fluids	34	13.55
Hair-feathers-skin	25	9.96
Several	19	7.56
Imaging and remote sensing	22	8.76
Urine	19	7.56
Invertebrates	6	2.39

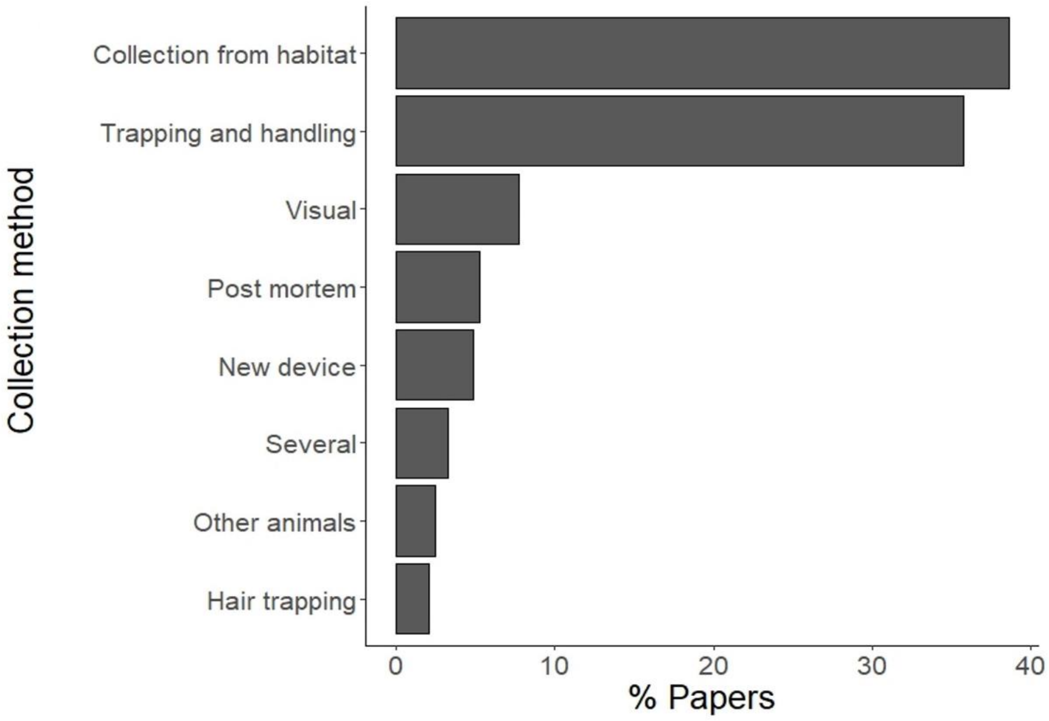

Figure S1. Percentage of published articles classified based on method used to collect samples.

Table S3. Number and percentage of papers published per biological material collection method (26 NA removed)

**Biological material collection method**	**N**	**%**
Collection from habitat	95	38.62
Trapping and handling	89	35.77
Visual	19	7.72
Post mortem	13	5.28
New device	12	4.87
Several	8	3.25
Other animals	6	2.43
Hair trapping	5	2.03

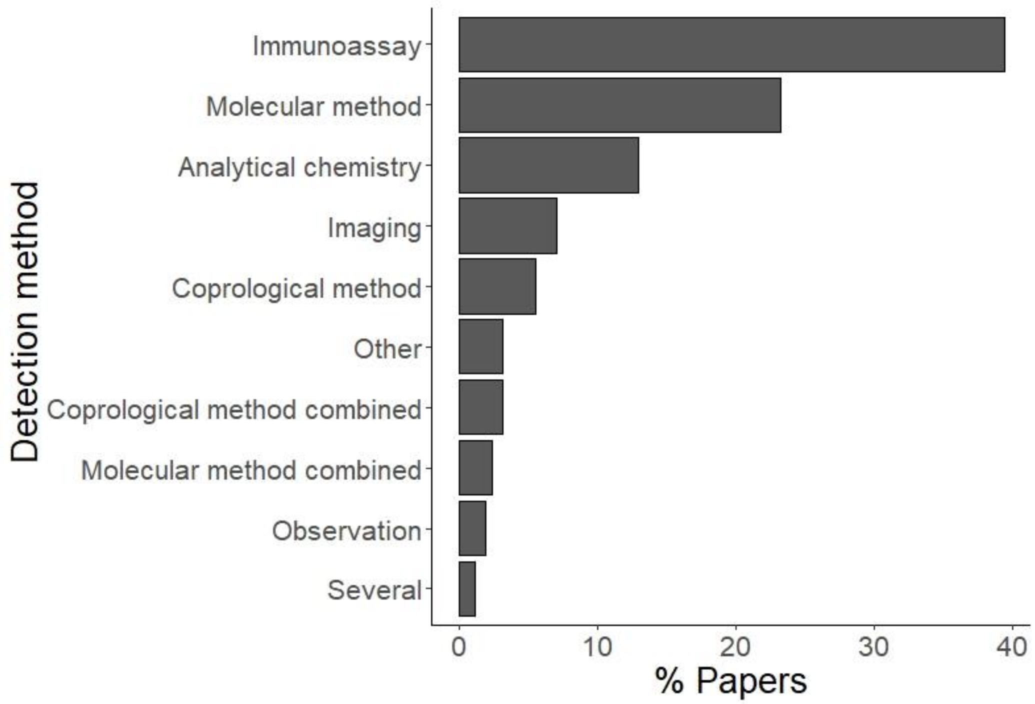

Figure S2. Percentage of published articles classified based on detection method.

Table S4. Number and percentage of papers published per detection method (18 NA removed).

**Detection method**	**N**	**%**
Immunoassay	100	39.37
Molecular method	59	23.22
Analytical chemistry	34	12.99
Imaging	18	7.09
Coprological method	14	5.51
Coprological method combined	8	3.15
Molecular method combined	6	2.36
Observation	5	1.96
Other	4	3.15
Several	3	1.18

Table S5. Percentage contribution to Chi-squared independence test biological material sample collected – Host's taxon.

	Amphibia	Aves	Fish	Mammalia	Marine mammal	Reptilia
**Faeces**	3.935	0.104	1.041	1.366	2.52	0.471
**Hair-feathers-skin**	0.974	3.046	0.195	0.003	0.909	0.112
**Imaging and remote sensing**	0.762	0.081	0.152	0.319	2.484	2.838
**Invertebrates**	0.254	0.356	0.051	0.037	0.237	2.661
**Saliva and other body fluids**	38.07	2.015	10.669	7.282	12.499	0
**Several**	0.762	0.081	0.152	0.11	0.001	0.356
**Urine**	0.019	1.126	0.161	0.663	0.751	0.375

Table S6. Percentage contribution to Chi-squared independence test Topic – Biological material sample collected.

	Faeces	Hair-feathers-skin	Imaging and remote sensing	Invertebrates	Saliva and other body fluids	Several	Urine
**Bacteria**	0.074	0.536	0.42	0.14	1.86	0.35	0.004
**Diet**	0	0.402	0.661	0.04	0.227	0.12	0.127
**Disease**	1.229	0.23	5.834	1.945	0.011	0.18	0.236
**Ectoparasites**	1.229	0.128	5.834	0.06	0.011	0.273	0.19
**Endoparasites**	1.509	0.307	0.24	0.08	0.453	0.24	0.253
**Fungi**	0.935	0.307	0.109	0.08	8.441	0.24	0.253
**Helminths**	1.645	0.728	0.57	0.19	1.076	0.05	0.068
**Immunity**	0.037	0.268	0.21	0.07	0.396	0.21	8.65
**Other**	0.037	0.066	0.21	1.571	0	0.175	0.222
**Physiology**	0.184	0.216	4.455	0.35	0.305	0.295	0.008
**Pollutants**	1.115	21.629	0.36	0.12	0.68	0.005	0.38
**Protozoa**	0.001	0.804	0.24	1.293	0.453	0.109	0.253
**Reproductive condition**	0.99	0.575	0.45	0.15	0.85	0.45	1.121
**Stress**	1.211	0.068	1.889	0.63	0.201	0.248	0.711
**Virus**	0.913	0.085	0.005	2.886	0.358	0.623	0.089
